# Unusual Tachycardia Association In A patient Without Structural Heart Disease

**Published:** 2009-07-01

**Authors:** Eduardo Arana-Rueda, Alonso Pedrote, Lorena Garcia-Riesco, Manuel Frutos-Lopez, Juan A Sanchez-Brotons

**Affiliations:** Arrhythmia Unit, Virgen del Rocío University Hospital, Seville, Spain

**Keywords:** Atrial flutter, Bundle branch reentry, Ventricular tachycardia, Ablation

## Abstract

We report an unusual association of persistent atrial flutter and bundle branch re-entrant ventricular tachycardia in a young patient without structural heart disease. Atrial flutter masked the infra-Hisian conduction disease, was fundamentally dependent on a long PR interval, and could be a possible trigger of ventricular tachycardia.

## Case Report

**Figure 1:**
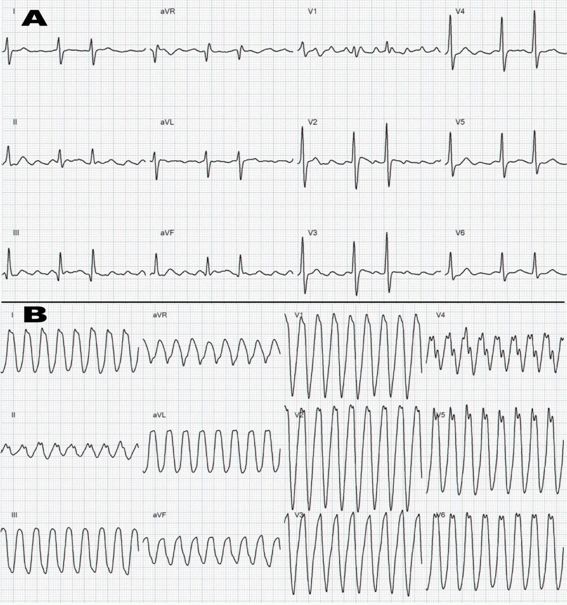
A. Basal ECG with atrial flutter and unspecific intraventricular conduction delay. B. Clinical Tachycardia with left bundle branch block, leftward axis and heart rate of 210 beats per minute.

A 28-year-old female with a recent diagnosis of persistent atrial flutter (AFL) and no structural heart disease, was undergoing treatment with betablocker while waiting for ablation (Figure 1A). She was admitted to the emergency department with palpitations on effort and recurrent syncope. The electrocardiogram (ECG) (Figure 1B) showed a wide QRS complex tachycardia with left bundle branch block, leftward axis and heart rate of 210 beats per minute, and she required direct electrical cardioversion because of poor clinical tolerance with severe hypotension. The ECG during sinus rhythm showed a first-degree atrioventricular (AV) block and non-specific intraventricular conduction delay. The AFL relapsed in the next hour after cardioversion so the treatment with betablocker alone was continued. All biochemical markers of myocardial ischaemia and electrolytes were within normal limits. The echocardiogram and cardiac magnetic resonance imaging showed no structural heart disease, with no chamber dilatation or hypertrophy and a left ventricular ejection fraction of 65%. With the clinical diagnosis of AFL with 1:1 AV conduction an electrophysiological study (EPS) was done in the next 72 hours.

**Figure 2:**
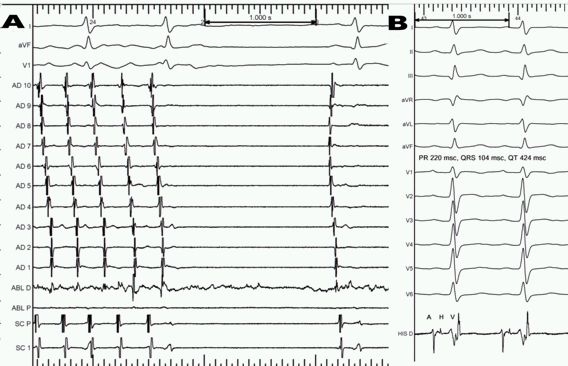
A: Finish of atrial flutter during ablation with block in the cavotricuspid isthmus. B: ECG after atrial flutter ablation with PR 220 msec, QRS 104 msec, HV 90 msec. AD1-10 duodecapolar catheter (AD1: Low lateral right atrial electrogram). His: His bundle electrogram. SC: coronary sinus.

**Figure 3:**
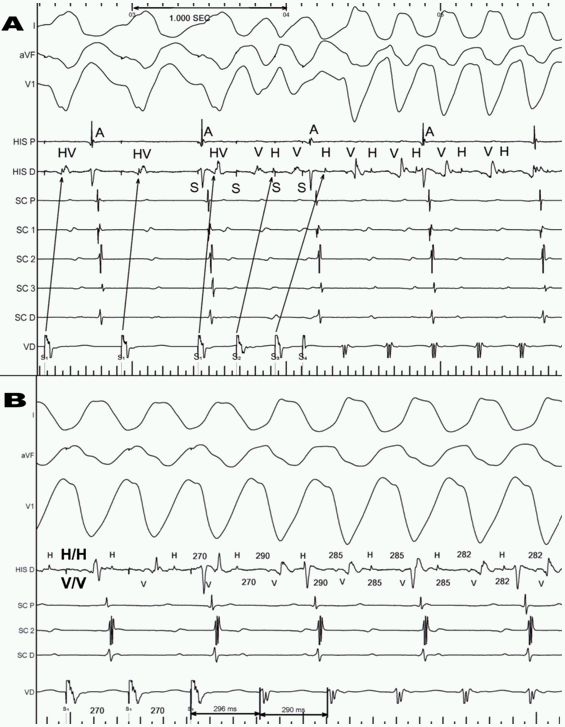
A. Initiation of clinical tachycardia with two right ventricular apex strastimuli (third does not capture). A critical V-H interval prolongation is necessary for the induction. B. Right ventricular apex entraintment with ECG fusion and post-pacing interval +6 msec. In A and B you can see AV dissociation, fixed HV interval during tachycardia longer than baseline and spontaneous changes in VV intervals preceded by similar changes in HH intervals.

During the EPS, the patient was in persistent AFL with a cycle length of 255 ms associated with a typical isthmus-dependent circuit. The AV conduction was variable with a HV interval 90 msec. Cavotricuspid isthmus ablation stopped the tachycardia (Figure 2A) restoring sinus rhythm with AH interval 78 msec and HV 90 msec (Figure 2B). After complete bidirectional isthmus block, we continued the EPS with programmed ventricular stimulation to complete the wide QRS tachycardia study protocol. With 2 extrastimuli we reproducibly induced the sustained clinical tachycardia. Diagnostic criteria for bundle branch re-entrant ventricular tachycardia (BBR VT) were typical ECG morphology, reproducible initiation of tachycardia with critical V-H interval prolongation, fixed HV interval longer than baseline, spontaneous changes in VV intervals preceded by similar changes in HH intervals and right ventricular apex entrainment with fusion and a post-pacing interval of <20 msec (Figure 3A and B). After ablation of the right bundle branch no induction of the clinical tachycardia or other arrhythmias were possible. The HV interval then was 92 msec, so definitive cardiac pacing was not required. She has remained asymptomatic without medical treatment at 6 months' follow-up.

## Discussion

BBR VT is often encountered in patients with dilated cardiomyopathy and with less frequency in valvular, ischemic and other structural heart disease, but is rarely seen in patients with a normal heart [1]. The tachycardia cycle length is typically short (<300 msec) and poorly tolerated hemodynamically, causing recurrent syncope or even sudden death. His-Purkinje system disease, frequently evident with long PR interval and/or prolonged QRS, is necessary in the genesis of arrhythmia, and during EPS virtually all patients have a long HV interval at baseline or after His-Purkinje system stress manoeuvres [2].

In our patient, without structural heart disease or bundle branch block and with persistent AFL, His-Purkinje system disease could be suspected only by a mild non-specific intraventricular conduction delay. This is highly unusual, the absence of the typical clinical and ECG features in this setting makes it difficult to consider the final diagnosis, and invasive testing is essential. Several reports have related BBR VT induction with atrial stimulation as well as with AFL/fibrillation [3]. We suppose that the coincidence in time between recurrent syncope and persistent AFL can be explained because AFL acted like a trigger for BBR VT.Several mechanisms of wide-QRS-complex tachycardia have to be ruled out before a BBR VT diagnosis can be established [1]. Myocardial VT and AV nodal tachycardias should be particularly considered when VA dissociation is present. Initiation of tachycardia with critical V-H interval prolongation suggests that induction of the tachycardia depends on conduction delay within the His-Purkinje system. Pacing maneuvers can be extremely helpful in identifying the bundle branch reentry mechanism when stable recording of the His, RB or LB potentials during VT are not possible. Ability to dissociate His or RB potential would strongly argue against bundle branch reentry mechanism. The combination of concealed entrainment (concealed QRS fusion) by atrial pacing and manifest entrainment (manifest QRS fusion) by ventricular pacing has been proposed as a useful diagnostic criterion for BBR VT with LBBB QRS morphology [4]. The difference between the first postpacing interval after right ventricular apex VT entrainment and the tachycardia cycle length can be used to rapidly screen for VT mechanism. As the right bundle inserts in the right ventricular apex, BBR VT is unlikely if the difference is longer than 30 ms [5].In our case, catheter ablation allowed complete control of arrhythmias with no device required because left ventricular ejection fraction was normal and HV interval was <100 msec.Abnormalities of the genes encoding cardiac ion channels have been found to be an important etiologic factor in some cases of conduction disturbance with tachyarrhythmias of different origin in normal hearts. Mutations of SCN5A-encoding cardiac sodium channel cause inherited susceptibility to ventricular arrhythmias (congenital long-QT syndrome, idiopathic ventricular fibrillation), cardiac conduction disease, and dilated cardiomyopathy with atrial arrhythmia [6]. It may also be present with more complex phenotypes; some cases reported multiple familial disorders manifested with congenital diffuse electrical disease in children with normal heart, sick sinus syndrome, intraventricular conduction block and monomorphic ventricular tachycardia [7]. Future studies must characterize the relationship between the SCN5A genotype, polymorphisms and cardiac phenotype. Long-term follow-up might clarify a possible association with an incipient cardiomyopathy or diffuse conduction heart system disease in our patient.
